# Selective digestive decontamination and antimicrobial resistance: defining thresholds before global implementation

**DOI:** 10.1128/aac.00681-26

**Published:** 2026-07-21

**Authors:** Shubhendu Bajpai, Abdullah Sultany, Kevin Carroll, Rahul Zain, Apurwa Karki

**Affiliations:** 1 Department of Internal Medicine, Guthrie Robert Packer Hospital 23128, Sayre, Pennsylvania, USA; 2 Department of Pulmonary and Critical Care Medicine, Guthrie Robert Packer Hospital 23128, Sayre, Pennsylvania, USA; Houston Methodist Hospital and Weill Cornell Medical College, Houston, Texas, USA

**Keywords:** low- and middle-income countries, Surviving Sepsis Campaign, multidrug-resistant organisms, sepsis, antimicrobial resistance, selective digestive decontamination

## LETTER

A recent point-prevalence study by Cornistein et al. involving 164 intensive care units (ICUs) in Argentina found that 45.5% of confirmed infections were attributable to multidrug-resistant organisms (MDROs), with carbapenem-resistant *Acinetobacter baumannii* and carbapenemase-producing Enterobacterales each representing 28.5% of MDRO isolates (1). These results are particularly relevant, given the 2026 Surviving Sepsis Campaign (SSC) guidelines, which conditionally recommend selective decontamination of the digestive tract (SDD) for mechanically ventilated adults with sepsis or septic shock in units characterized by a “low prevalence of antimicrobial resistance” (2). Although this recommendation is supported by a meta-analysis of 30 randomized controlled trials indicating a probable reduction in mortality (RR 0.91; 95% CI, 0.82–0.99) (3), the lack of a quantitative definition for “low resistance” raises significant concerns about resistance selection pressure in contexts similar to those described by Cornistein et al. (1).

Most evidence supporting SDD originates from ICUs in Australia, Canada, and the Netherlands, where baseline MDRO prevalence remains low (3, 4). Conversely, the R-GNOSIS trial, which included 13 European ICUs with moderate-to-high resistance (17.6% rectal carriage of third-generation cephalosporin-resistant Enterobacteriaceae), did not demonstrate a mortality benefit from decontamination strategies (5). In the absence of an explicit resistance benchmark, whether based on rectal carriage rates, bloodstream infection MDRO proportions, or unit-level antibiogram data, clinicians lack practical guidance to determine SDD eligibility for their ICUs. Establishing a supplementary framework with measurable thresholds and defined reassessment intervals would significantly improve the clinical applicability of this recommendation.

The SSC guidelines further state that “in low resource settings where prevalence of antimicrobial resistance is high and there is limited availability of antimicrobial therapy, SDD should be implemented on an ICU-by-ICU basis” (2). However, this guidance does not fully address the structural barriers present in low- and middle-income countries (LMICs), where antimicrobial resistance surveillance is frequently inadequate and many ICUs lack the microbiological capacity to produce reliable antibiograms (6). The SDD regimen includes polymyxins and aminoglycosides, which are considered last-resort therapies in many LMICs. Prophylactic use of these agents in settings with a high MDRO burden, as described by Cornistein et al., may further increase resistance selection among pathogens that are already challenging to treat (1, 7).

We recommend two essential actions: (i) the establishment of a consensus-based, quantitative definition of “low antimicrobial resistance prevalence” to inform SDD eligibility and (ii) the initiation of dedicated randomized controlled trials assessing SDD in LMIC ICUs with high MDRO prevalence, using resistance emergence as a primary endpoint. These measures are critical to ensure that SDD can be implemented safely and without worsening the global antimicrobial resistance crisis.
